# Full-length 16S rRNA amplicon sequencing reveals the variation of epibiotic microbiota associated with two shrimp species of Alvinocarididae: possibly co-determined by environmental heterogeneity and specific recognition of hosts

**DOI:** 10.7717/peerj.13758

**Published:** 2022-08-08

**Authors:** Min Hui, Aiyang Wang, Jiao Cheng, Zhongli Sha

**Affiliations:** 1 Department of Marine Organism Taxonomy & Phylogeny, Institute of Oceanology, Chinese Academy of Sciences, Qingdao, China,; 2 Laboratory for Marine Biology and Biotechnology, Qingdao National Laboratory for Marine Science and Technology, Qingdao, China,; 3 Shandong Province Key Laboratory of Experimental Marine Biology, Institute of Oceanology, Chinese Academy of Sciences, Qingdao, China,; 4 University of Chinese Academy of Sciences, Beijing, China

**Keywords:** Alvinocarididae, Deep-sea, Hydrothermal vent, Methane seep, Microbial diversity

## Abstract

Shrimps of the family Alvinocarididae, endemic species to deep sea chemosynthetic ecosystems, harbor epibiotic microbes on gills which probably play important roles in the survival of the shrimps. Among them, *Alvinocaris longirostris* and *Shinkaicaris leurokolos* occupy different ecological niches within the same hydrothermal vent in Okinawa Trough, and *A. longirostris* also exists in a methane seep of the South China Sea. In this study, full-length 16S rRNA sequences of the gill associated bacteria of two alvinocaridid species from different chemosynthetically ecological niches were first captured by single-molecule real-time sequencing. Totally, 120,792 optimized circular consensus sequences with ∼1,450 bp in length were obtained and clustered into 578 operational taxonomic units. Alpha diversity analysis showed seep *A. longirostris* had the highest species richness and evenness (average Chao1 = 213.68, Shannon = 3.39). Beta diversity analysis revealed that all samples were clearly divided into three groups, and microbial community of *A. longirostris* from seep and vent were more related than the other comparisons. By permutational multivariate analysis of variance, the most significant community compositional variance was detected between seep *A. longirostris* and vent *S. leurokolos* (*R*^2^ = 0.731, *P* = 0.001). The taxon tags were further classified into 21 phyla, 40 classes, 89 orders, 124 families and 135 genera. Overall, the microbial communities were dominated by Campylobacteria and Gammaproteobacteria. Alphaproteobacteria, Bacteroidia, Verrucomicrobiae, Bacilli and other minor groups were also detected at lower abundance. Taxonomic groups recovered from the vent *S. leurokolos* samples were only dominated by Sulfurovaceae (94.06%). In comparison, gill-associated microbiota of vent *A. longirostris* consisted of more diverse sulfur-oxidizing bacteria, including Sulfurovaceae (69.21%), Thiotrichaceae (6.77%) and a putative novel Gammaproteobacteria group (14.37%), while in seep *A. longirostris*, Gammaproteobacteria un-group (44.01%) constituted the major component, following the methane-oxidizing bacteria Methylomonadaceae (19.38%), and Sulfurovaceae (18.66%). Therefore, the gill associated bacteria composition and abundance of alvinocaridid shrimps are closely related to the habitat heterogeneity and the selection of microbiota by the host. However, the interaction between these alvinocaridid shrimps and the epibiotic communities requires further study based on metagenome sequencing and fluorescence *in situ* hybridization.

## Introduction

Deep-sea hydrothermal vents and cold seeps are chemosynthetic ecosystems characterized by high pressure, low oxygen, absence of light, and high concentration of various chemicals (McMullin, Bergquist & Fisher, 2007). Despite these similarities, there are also extensive differences between vents and seeps. Hydrothermal vents are mainly distributed in mid-ocean ridges, volcanic arcs, and back-arc spreading centers, which are usually ephemeral without strong sedimentation (Beaulieu et al., 2013). In contrast, cold seeps are characterized by thicker sediment and discharge of low-temperature hydrocarbon-rich fluids to the seafloor along passive continental margins and subduction zones (Sibuet & Olu, 1998). Moreover, vents are usually richer in hydrogen sulfide and heavy metals than those of seeps (Cosson & Vivier, 1997; Miyazaki et al., 2013). In addition, the temperature of the hydrothermal fluid can reach over 400 °C, and a steep thermal gradient is specifically created by mix of the vent fluid and the surrounding seawater (∼2 °C) (Koschinsky et al., 2008; Zhu et al., 2020), while in seeps, temperatures do not vary appreciably from those of the ambient deep-sea water.

In these extreme deep-sea hydrothermal vent and cold seep ecosystems, organisms rely on the free-living and symbiotic microbial chemosynthetic primary production (Dubilier, Bergin & Lott, 2008). Since Cavanaugh et al. (1981) first discovered the symbiotic relationship in tube worms from deep-sea hydrothermal vents at the Galapagos Rift and East Pacific Rise, similar symbiotic relationships have been found among other invertebrates living in deep-sea chemosynthetic ecosystems (Cary et al., 1997; Peek et al., 1998; Duperron et al., 2007; Saito, 2008). The innovative establishment of symbiotic relationships with chemosynthetic bacteria in invertebrates is one of the important factors that enable them to thrive in the extreme habitats (Petersen et al., 2012). It is now becoming increasingly clear that these symbionts play critical roles in supplying nutrition to their hosts (Rinke et al., 2006; Suzuki et al., 2009), detoxifying sulfides and heavy metals (Alayse-Danet, Desbruyeres & Gaill, 1987; Prieur et al., 1990; Grote et al., 2012), and protecting hosts from fungi, pathogens and predators (Gil-Turnes, Hay & Fenical, 1989; Gil-Turnes & Fenical, 1992; Goffredi et al., 2004; Lopanik, Lindquist & Targett, 2004; Lindquist, Barber & Weisz, 2005).

Shrimps of the family Alvinocarididae are endemic species to chemosynthetic ecosystems. It has been revealed that the microbes of alvinocaridid species mainly colonize gill chamber and gut (Hügler et al., 2011; Sun et al., 2016). The best research example of alvinocaridid species is *Rimicaris exoculata* from the Mid Atlantic Ridge. Recent studies have demonstrated that *R. exoculata* epibiotic communities consist of two major groups, Campylobacteria (previously Epsilonproteobacteria) and Gammaproteobacteria, and lower abundances of Deltaproteobacteria, Alphaproteobacteria, Zetaproteobacteria, Betaproteobacteria, and Bacteroidetes (Zbinden et al., 2008; Petersen et al., 2010; Hügler et al., 2011; Guri et al., 2012; Jan et al., 2014; Cambon-Bonavita et al., 2021). It has been evidenced that the chemosynthetic ectosymbionts of *R. exoculata* can directly transfer nutrients to the host (Ponsard et al., 2013). Sulfide, methane, iron and hydrogen oxidation have been proposed occurring within this community (Zbinden et al., 2004; Corbari et al., 2008a; Corbari et al., 2008b; Zbinden et al., 2008; Petersen et al., 2011; Hügler et al., 2011; Jan et al., 2014; Cambon-Bonavita et al., 2021). Therefore, the high metabolic diversity indicates that the epibiotic microbial community of *R. exoculata* is variable, which may facilitate the shrimp thriving in the dynamic hydrothermal mixing zones (Cambon-Bonavita et al., 2021) and colonizing geochemically different hydrothermal vents along the Mid-Atlantic Ridge (Zbinden & Cambon-Bonavita, 2020). However, limited studies on microbial associated with other species from Alvinocarididae have been conducted (Tokuda et al., 2008; Sun et al., 2016; Apremont et al., 2018), and their diversity of epibiotic communities remains largely unknown.

**Figure 1:**
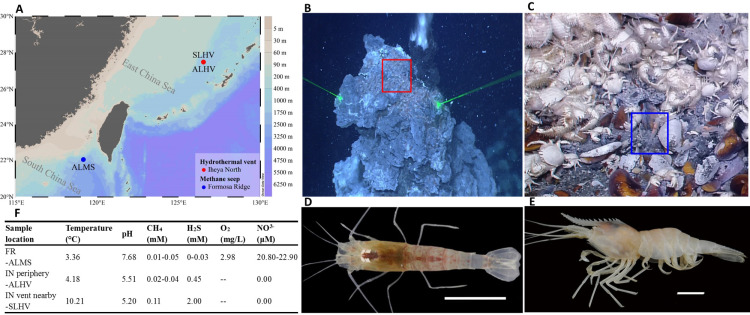
Sampling information of alvinocaridid shrimps. (A) Sampling sites of alvinocaridid shrimps. The map was created by Ocean Data View (ODV) v5.3.0.0 (Schlitzer, 2020). Different colors on the right color bar represent different water depths. ALMS: *A. longirostris* from Methane Seep; ALHV: *A. longirostris* from Hydrothermal Vent; SLHV: *S. leurokolos* from Hydrothermal Vent. (B) The *S. leurokolos* living environment in the Iheya North hydrothermal vent of Okinawa Trough. The *S. leurokolos* is marked in the red square. (C) The *A. longirostris* living environment in the Formosa Ridge methane seep of the South China Sea. The *A. longirostris* is marked in the blue square. (D) Morphological characteristics of *S. leurokolos*. (E) Morphological characteristics of *A. longirostris*. (F) Environmental parameters of the living environment of different sample groups. FR: Formosa Ridge; IN: Iheya North.

In the Okinawa Trough, it has been found that different species of alvinocaridid shrimps occupy different ecological niches within the same hydrothermal vent ecosystem. *Shinkaicaris leurokolos* inhabits the area close to the hydrothermal vent (Figs. 1B & 1D), while *Alvinocaris longirostris* inhabits on the mussel bed that surrounds the base of hydrothermal vents. Moreover, *A. longirostris* is the only alvinocaridid shrimps that has been found in both hydrothermal vent and cold seep areas (Figs. 1A, 1C & 1E; (Tokuda et al., 2006). Considering the differences in physical and geochemical conditions of hydrothermal vents and cold seeps, as well as the thermal and chemical gradients within the vent environments, it has been hypothesized that these shrimps inhabiting different chemosynthetic environments may differ in diversity of epibiotic microbial communities.

Hence, this study aimed to test the hypothesis by (1) obtaining full-length 16S rRNA sequences of microbiota on the gill of different alvinocaridid shrimps using single-molecule real-time (SMRT) sequencing, (2) characterizing and comparing the composition and structure of gill microbial community of shrimps inhabiting different chemosynthetic ecosystems (hydrothermal vent *vs* methane seep), and different ecological niches (near the vent *vs* periphery of vent) within the same hydrothermal vent, (3) determining the main factors influencing the microbial diversity.

## Materials & Methods

### Sampling

The alvinocaridid shrimps were collected during the cruise by the scientific research vessel (RV) KEXUE (Institute of Oceanology, Chinese Academy of Sciences, China) in July 2018. Within the Iheya North hydrothermal vent (the depth about 1000 m) area of Okinawa Trough, shrimps *S. leurokolos* were sampled around the active hydrothermal chimney wall (Figs. 1A and 1B; 126°53.80′E, 27°46.46′N), and abbreviated hereafter to SLHV (*S. leurokolos* from Hydrothermal Vent), while *A. longirostris* were taken from the periphery of the vent (126°53.86′E, 27°47.02′N) and abbreviated hereafter to ALHV (*A. longirostris* from Hydrothermal Vent) (Fig. 1A). Meanwhile, another group of *A. longirostris* was collected from the Formosa Ridge methane seep (119°17.14′E, 22°06.93′N, 1119.20 m deep) in the South China Sea and abbreviated hereafter to ALMS (*A. longirostris* from Methane Seep) (Figs. 1A and 1C). Samples were taken by the remotely operated vehicle (ROV) in the RV KEXUE. Once aboard, the gill tissues were dissected and immediately frozen in liquid nitrogen. Finally, it was stored at −80 °C until use.

### DNA extraction and SMRT sequencing

Total genomic DNA from 10 shrimp gills (three ALMSs, three ALHVs, and four SLHVs) was extracted by TGuide S96 Magnetic Soil /Stool DNA Kit (TIANGEN Biotech, Beijing, China) according to the manufacturer’s instruction, respectively. In addition, DNA concentration of the sample was measured with the Qubit dsDNA HS Assay Kit and Qubit 4.0 Fluorometer (Invitrogen, Thermo Fisher Scientific, Waltham, MA, USA). Polymerase Chain Reaction (PCR) amplification of the nearly full-length bacterial 16S rRNA genes was performed using the bacteria-specific primers integrated with symmetric barcodes: 27F (5′-Barcode- AGRGTTTGATYNTGGCTCAG-3′) and 1492R (5′-Barcode-TASGGHTACCTTGTTASGACTT-3′). The KOD One PCR Master Mix (TOYOBO Life Science) was used to perform PCR amplification, and the PCR cycling program was set as follows: denaturation at 95 °C for 5 min, 25 cycles of 95 °C for 10 s, 55 °C for 30 s and 72 °C for 90 s and final elongation at 72 °C for 2min. Amplicons were subsequently quantitatively analyzed using agarose gel electrophoresis and digital analysis by ImageJ (Abràmoff, Magalhães & Ram, 2004). Equimolar samples were mixed and purified with Agencourt AMPure XP Beads (Beckman Coulter, Indianapolis, IN, USA) and quantified using the Qubit dsDNA HS Assay Kit and Qubit 4.0 Fluorometer (Invitrogen, Thermo Fisher Scientific, Oregon, USA). SMRT bell libraries were prepared from the amplified DNA by SMRT bell Express Template Prep Kit 2.0 according to the manufacturer’s instructions (Pacific Biosciences). Purified SMRT bell libraries from the pooled and barcoded samples were sequenced on a single PacBio Sequel II 8M cell using the Sequel II Sequencing kit 2.0. The raw reads generated from sequencing were filtered and demultiplexed using the SMRT Link software (version 8.0) with the minPasses ≥3 and minPredictedAccuracy ≥0.90 in order to obtain the circular consensus sequences (CCS). Subsequently, the lima v1.7.0 was employed to assign the CCS to the corresponding samples based on their barcodes, and the CCS that did not contain primers were removed by Cutadapt v2.7. The sequences outside the expected size (<1340 and >1640 bp) were further filtered out, and the chimeric sequences with two or more segments were identified and deleted by UCHIME version 10.0 (Edgar et al., 2011). Finally, high-quality optimized CCS were generated for the downstream analysis.

### Statistical analyses

The effective reads were clustered into operational taxonomic units (OTUs) at 97% similarity level according to Tindall et al. (2010) and Pootakham et al. (2021) by USEARCH (Edgar, 2010). The RDP Classifier (Wang et al., 2007) was used to classify representative sequences of each OTU into different taxonomic groups based on the Silva database (release 138) (Quast et al., 2012).

Alpha diversity (within samples) was computed at the OTU level using the Mothur v1.30 (Schloss et al., 2009), including the number of OTUs, Chao1 estimate of species richness, Shannon diversity, Good’s coverage and rarefaction curve, which were displayed with R software. To examine the Beta diversity (among samples), principal coordinates analysis (PCoA) was performed based on unweighted unifrac at the OTU level using the QIIME (Caporaso et al., 2010). The statistically significant differences of Chao1 and Shannon among groups were analyzed through Wilcoxon rank-sum test in agricolae package. To further compare the bacterial communities in different samples, permutational multivariate analysis of variance (PERMANOVA) was performed with the package vegan in R software.

### Phylogenetic analyses

In order to study the phylogenetic relationship of different OTUs and the difference of the dominant species in different groups, sequences of OTUs with cumulative percentage 95% in each group (ALMS, ALHV, SLHV) and their close relatives retrieved from the Silva database were aligned using MAFFT v7.475 (Katoh & Standley, 2013). The best-fitting model of sequence evolution was selected by ModelFinder, and maximum-likelihood (ML) phylogenetic tree with 1,000 bootstrap replicates was reconstructed using IQ-TREE v2.1.2 (Kalyaanamoorthy et al., 2017). Tree graphics was viewed, manipulated and annotated with the FigTree v.1.4.4 software (http://tree.bio.ed.ac.uk/software/figtree/).

## Results

### SMRT high-throughput amplicon sequencing of full-length 16S rRNA gene

**Figure 2:**
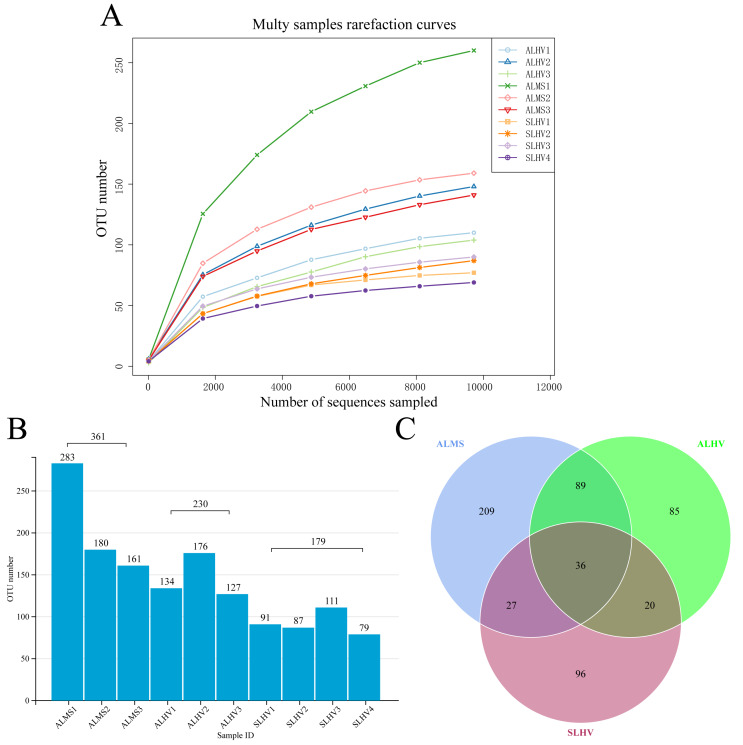
Operational taxonomic units (OTUs) of different samples. (A) Rarefaction curve diagrams of OTUs from samples of ALHV, ALMS, and SLHV groups. (B) OTU number distribution in each sample and groups. (C) Venn diagram of OTUs among the three groups.

Full-length 16S rRNA genes were amplified and sequenced from bacterial communities associated with gills of *S. leurokolos* and *A. longirostris* collected from different sampling sites (Fig. 1A). A total of 123,022 raw reads were obtained from the 10 gill samples (Table 1), with an average of 12,302 sequences per sample. The raw data was deposited in FigShare (https://figshare.com/s/bf5c74b5a9519c981a1b) and NCBI SRA database (PRJNA791844). After quality control, filtering, and removing the chimeras, 120,792 optimized CCS with approximately 1,450 bp in length were retained and used for subsequent analysis. Based on 97% sequence similarity, effective tags were clustered into 568 OTUs. The rarefaction curve for the number of OTUs almost approached saturation in each sample (Fig. 2A), suggesting that most of the 16S rRNA gene sequences associated with the samples were covered by the sequencing data. The Good’s coverage values were above 0.99 for all samples, agreeing with the rarefaction analysis that overall bacterial diversity was encompassed by our sequencing effort (Table 1).

### Diversity of gill associated microbiota in different alvinocaridid shrimps

**Figure 3:**
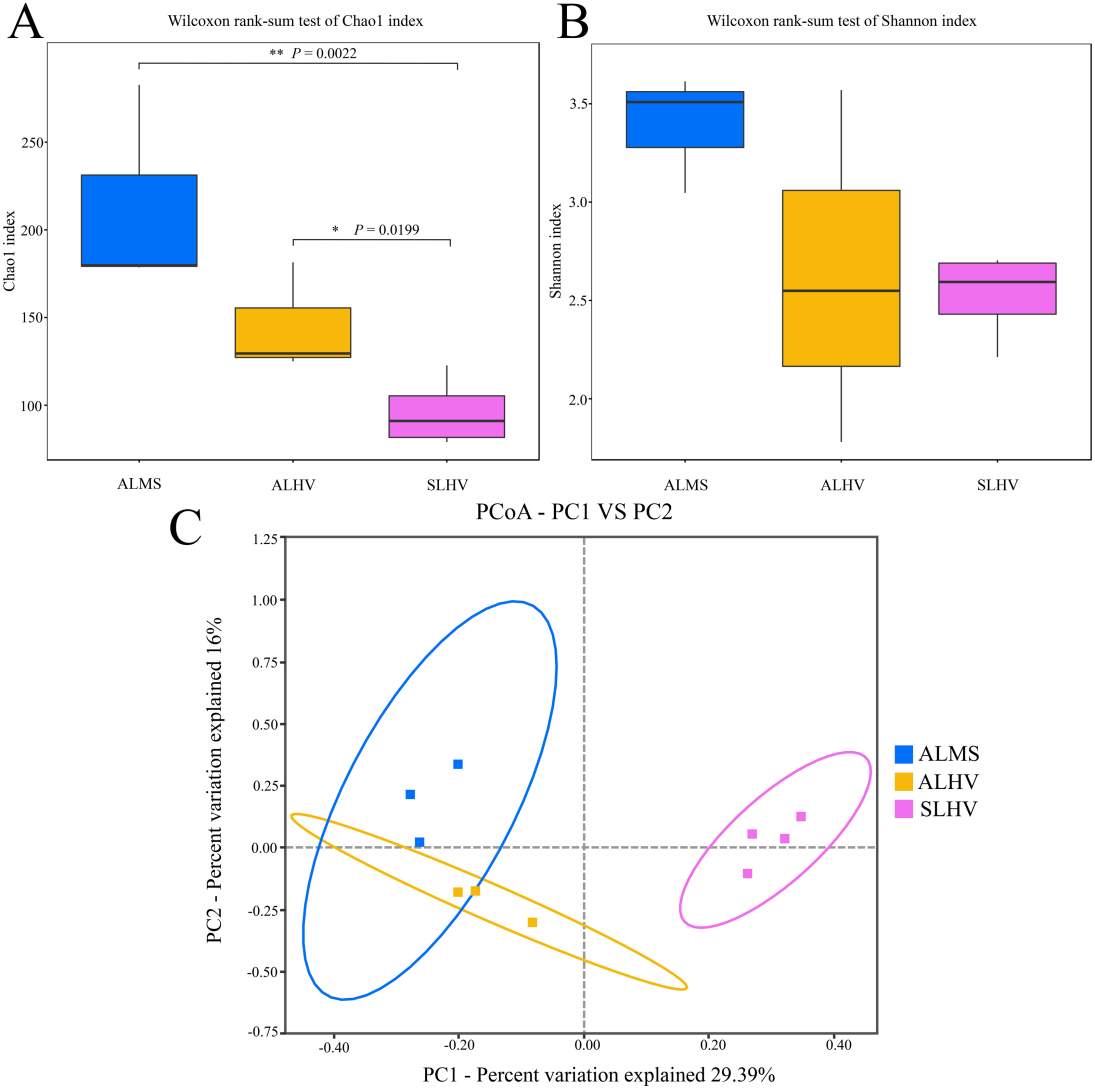
Microbiota alpha and beta diversity evaluated based on the Chao1 (A), Shannon (B) indices, and principal coordinates analysis (PCoA) among ALMS, ALHV, and SLHV (C). An asterisk (*) indicates significant differences with *P* < 0.05; two asterisks (**) indicates extremely significant difference with *P* < 0.01.

To assess the diversity of bacterial community within each sample and each group, a series of alpha diversity indices were calculated. It was revealed that ALMS samples had the highest number of OTUs (283), while the lowest (93) was found in SLHV samples (Fig. 2B). As shown by the Venn diagram (Fig. 2C), 36 OTUs were shared among the three groups, and 209, 85 and 96 unique OTUs were identified in ALMS, ALHV, and SLHV, respectively. Therefore, the bacterial assemblages on gill of *A. longirostris* from the methane seep environment are more diverse than those of samples from vent area based on OTU number, and however, the three groups still shared some OTUs regardless of the geographic distance and their environmental differences. The Chao1 index, as richness index of bacterial community, showed a corresponding trend with OTU numbers. The highest species richness appeared in ALMS samples (average Chao1 = 213.68), followed by ALHV (145.33), and the lowest was SLHV (95.97) (Table 1). Shannon index takes into account not only the richness but also the evenness of the community. Samples of ALMS also showed the highest average Shannon index (3.39), while SLHV exhibited the lowest Shannon value (2.53). By statistics (Figs. 3A and 3B, Table S1), it was found that the difference of Chao1 between ALMS and SLHV was extremly significant (*P* < 0.01), as well as ALHV and SLHV (*P* < 0.05), while there was no significant difference between ALHV and ALMS (*P* > 0.05). However, no significant difference of Shannon index was detected in any comparison. These results indicate that the bacterial community composition between ALHV and ALMS is more similar than the other two comparisons (ALMS *vs* SLHV, and ALHV *vs* SLHV).

**Table 1:** Summary of circular consensus sequences (CCS), operational taxonomic units (OTUs), Good’s coverage and alpha diversity indices information.

Samples	Barcode CCS number	Optimized CCS number	OTU number	Good’s coverage	Chao1	Shannon
ALMS1	12,987	12,503	283	0.994	282.65	3.61
ALMS2	10,338	10,124	180	0.997	179.78	3.51
ALMS3	13,195	12,966	161	0.996	178.63	3.05
**Average**	12,173	11,864	208	0.996	213.68	3.39
ALHV1	11,726	11,608	134	0.997	125.04	2.55
ALHV2	13,003	12,823	176	0.996	181.44	3.57
ALHV3	13,050	12,847	127	0.997	129.50	1.78
**Average**	12,593	12,426	146	0.997	145.33	2.63
SLHV1	12,968	12,739	91	0.999	82.57	2.21
SLHV2	10,020	9,877	87	0.997	122.77	2.70
SLHV3	12,749	12,529	111	0.998	99.55	2.69
SLHV4	12,986	12,776	79	0.998	79.00	2.50
**Average**	12,181	11,980	92	0.998	95.97	2.53
**Total**	123,022	120,792	568	–	–	–

Moreover, PCoA was performed to measure beta diversity of the three groups. It showed that all gill samples were clearly divided into three clusters, among which ALMS and ALHVgroups were more related than the others (Fig. 3C). It also indicated that ALHV and ALMS groups shared similar microbiota, while SLHV group was different with the others in the gill microbiota. The differences of microbial community composition among groups of ALMS, ALHV, and SLHV were further confirmed with PERMANOVA (Table S2). The community compositional variance between ALMS and SLHV was significant (*R*^2^ = 0.731, *P* = 0.001), as well as ALHV *vs* SLHV comparison (*R*^2^ = 0.862, *P* = 0.037), while no difference between ALMS and ALHV was detected (*R*^2^ = 0.537, *P* = 0.100).

### Microbial community at different taxonomic level

Using a confidence threshold of 80%, 114,938 out of 120,792 filtered reads were assigned using the RDP classifiers, which covered 21 phyla, 40 classes, 89 orders, 124 families, and 135 genera (Table S3).

**Figure 4:**
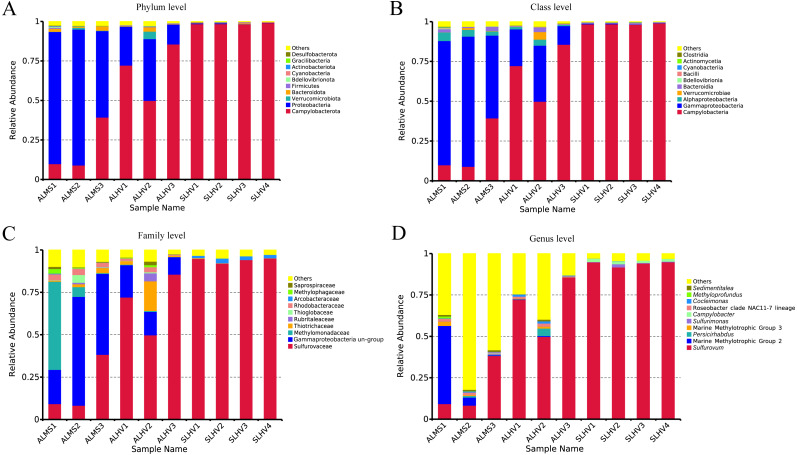
Relative abundances of bacteria at the phylum (A), class (B), family (C) and genus (D) level in gill samples of ALMS, ALHV, and SLHV.

At the phylum level, Campylobacterota (formerly Epsilonproteobacteria) (Waite et al., 2017; Waite et al., 2018; Parks et al., 2018) and Proteobacteria were the most abudant in all samples. Bacteroidota, Verrucomicrobiota, Bacteroidetes, Firmicutes, Bedellovibrionota and other minor groups were also detected at lower abundance (Fig. 4A). The main phylum in ALMS and ALHV were Proteobacteria (74.77% in ALMS *vs* 25.38% in ALHV) and Campylobacterota (19.45% in ALMS *vs* 69.29% in ALHV) (Table S4). In SLHV group, the single most abundant bacterial phylum was Campylobacterota (98.57%), which was consistently predominant in all four samples with relative abundance close to 100% (Fig. 4A; Table S4). At the class level, ALMS was dominated by Gammaproteobacteria (70.58%), followed by Campylobacteria (19.45%), Alphaproteobacteria (4.14%) and Bacteroidia (1.78%), while in ALHV, Campylobacteria (69.26%) took the largest proportion and others included Gammaproteobacteria (23.34%), Alphaproteobacteria (2.05%), Verrucomicrobiae (1.58%) and Bacteroidia (1.11%) (Fig. 4B; Table S4). In SLHV, most microbes belonged to Campylobacteria (98.56%).

Deep insight into the bacterial communities were obtained at the family level. It was shown that the ALMS group was dominated by an unidentified group belonging to Gammaproteobacteria (44.01%, abbreviated hereafter to Gammaproteobacteria un-group), Methylomonadaceae (19.38%), Sulfurovaceae (18.66%), Rhodobacteraceae (3.27%), Thiotrichaceae (1.77%), Thioglobaceae (1.69%) and Methylophagaceae(1.10%) which accounted for 89.88% of all sequences (Fig. 4C; Table S5). In the ALHV group, the dominant families were Sulfurovaceae (69.21%), Gammaproteobacteria un-group. (14.37%), Thiotrichaceae (6.77%), Rhodobacteraceae (1.70%), and Rubritaleaceae (1.57%), accounting for 93.62% of all sequences (Fig. 4C; Table S5). Taxonomic groups recovered from the SLHV samples were dominated by Sulfurovaceae (94.06%, Fig. 4C; Table S5) and Arcobacteraceae (2.11%). Overall, the gill associated bacterial taxomic compositions between ALMS and ALHV were similar but varied in abundance, while in SLHV, Sulfurovaceae was the absolutely dominant group.

### Phylogenetic analysis at low taxonomic level

**Figure 5:**
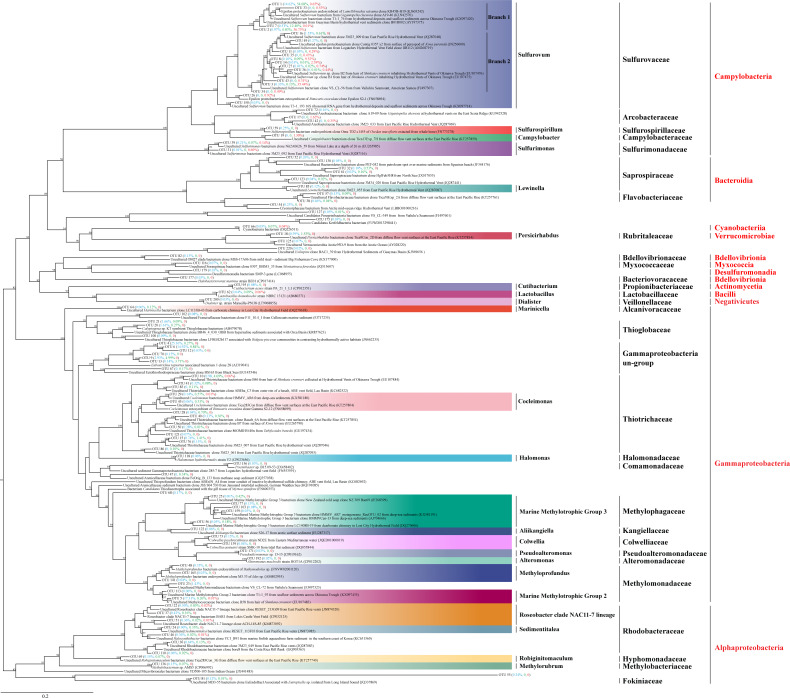
Phylogenetic analysis of the top OTUs. The phylogenetic tree is constructed based on 16S rRNA full-length sequences with the distance-based maximum-likelihood (ML) method using IQ-TREE. Numbers in the nodes correspond to ML bootstrap proportions (>50). Red, green and blue numbers in brackets represent relative abundances of the OTUs in ALMS, ALHV, and SLHV, respectively.

In total, 86,393 sequence tags (71.52%) were assigned to known genera including members of sulfur oxidizing or reducing bacteria, such as *Sulfuricurvum*, *Sulfurimonas*, *Sulfurospirillum*, and members of methylotropher, *Methyloprofundus* and *Methylorubrum*. *Roseobacter*, *Campylobacter* and *Cocleimonas* were also detected. Finally, 23,488 sequence tags (19.44%) were assigned to species, most of which were highly related (identities 97% to 100%) to the uncultured bacterium clones from chemosynthetic systems including sediments, fluids, and megafauna symbionts (Fig. 5; Table S6). Phylogenetic analysis of the 101 dominant OTU sequences were performed (Fig. 5), which constituted 95% of the sequence tags of each group (ALMS, ALHV, SLHV).

Among them, 16 OTUs were assigned to Sulfurovaceae all belonging to the genus *Sulfurovum*. OTU1, OTU2, OTU7 were clustered into one clade (Branch 1) and dominant in the three sample groups (Fig. 5; Table S6). OTU1, constituting 14.62%, 54.68% and 0.65% sequence tags in ALMS, ALHV and SLHV respectively, showed high identity (99.02%) to the uncultured *Sulfurovum* bacterium clone from hydrothermal deposits and seafloor sediments across the Okinawa Trough (KX097420). OTU7 constituting 12.40% of the sequence tags in ALHV and only dominant in this group, matched an uncultured *Sulfurovum* bacterium from the vent snail *Gigantopelta chessoia* clone (KU942570) with similarity 97.37%, while OTU2 belonging to *Sulfurovum*, took a high proportion (37.73%) in SLHV. However, OTU2, OTU8, OUT11, OTU14 on another branch (Branch 2) of *Sulfurovum* only showed high abundance (52.93% in total) in SLHV (Fig. 5; Table S6), which might be associated with the host seclection for bacteria or the extreme environment of vent nearby.

Many members of Thiotrichaceae and Thioglobaceae are sulfur-oxidizer. OTU10 and OTU15 accounted for 4.69% and 1.41% of sequence tags in ALHV resepectively, which were both assigned to Thiotrichaceae. OUT20 and OTU21 were assigned to Thioglobaceae, which constituted 3.27% sequence tags in ALMS. It was worth noted that OTU4 (25.16%) and OTU6 (14.92%) showed high abandance (in total 40.08%) in ALMS, which shared very low similarity percentage (<90%) of the 16S sequence with its relative close phylogenetic species *Eubostrichus topiarius* associated bacterium 1 (AJ319041) (Table S6). Within the same cluster, OTU9, OTU13, OTU67 and OUT70 also showed low identity (<91%) with its relatively close type strains, and OTU9 and OTU13 took the proportions of 4.99% and 3.71% in sequence tags of ALHV. The phylogenetic position of this cluster (including OTUs 4, 6, 9, 12, 13, 67, 70) located between the family Ectothiorhodospiraceae and Thioglobaceae, suggesting that the uncultured gill associated strains represented by these OTUs were potentially new species belonging to Gammaproteobacteria and related to sulfur metabolism.

In addition, relatively high abundance of methylotrophic related bacteria were identified in ALMS, including Methyloprofundus, Methylotrophic Group 2 and Marine Methylotrophic Group 3. Among them, OTU5 assigned to the Marine Methylotrophic Group 2, belonged to Methylomonadaceae and accounted for a high proportion (17.31%) of sequence tags from ALMS.

## Discussion

### The advantage of 16S rRNA full-length amplicons in revealing microbial hidden diversity

The microbial communities associated with megafauna in chemosynthetic ecosystems are believed to provide continuous nutrition to their hosts (Sogin et al., 2021). In the past years, the next-generation sequencing platforms have been widely used in studies of bacteria associated with faunas in chemosynthetic environments (Forget & Kim Juniper, 2013; Zwirglmaier et al., 2015; Sun et al., 2016; Tian et al., 2017; Zhang et al., 2018), but most of them use short reading amplicons which may not classify the microbes accurately at genus or species level. Especially when applied to the environment with a large number of uncultured bacteria, such as hydrothermal vents and cold seeps, this shortcoming will be magnified (Schloss et al., 2016). Prior to our study, although Sun et al. (2016) has attempted to discuss the bacterial community structure associated with *A. longirostris* from vent area at the genus level, only 4% of the sequence tags were assigned to known genera. The generation of SMRT sequencing allows producing an average length of more than 10 kb reads, which ensures the obtaining of complete 16S rRNA gene amplicon sequences. In our study, the gill associated bacteria diversity of different alvinocaridid shrimps from different hydrothermal vent and methane seep in the North Pacific has been revealed based on complete 16S sequences for the first time. More than 70% sequence tags have been classified at genus level, and approximately 20% tags can be assigned to species. Up further investigation, nine different OUTs belonging to *Sulfurovum* in this study have been aligned (Fig. S1), and it obviously shows that large amounts of variation will be omitted using V3-V4 or V4-V5 regions of 16S rRNA alone, which may hide the microbial biodiversity in the analysis. Therefore, our results highly improve the classification resolution at low taxonomical level and provide novel resources for genetic database of the deep-sea chemosynthetic life. As the improvement of SMRT high-throughput accuracy, the 16S rRNA full-length sequences based on the third-generation sequencing are expected to be more widely applied in the studies of microbial community in typical marine ecosystems (Pootakham et al., 2017; Sun et al., 2020).

Moreover, sequence and phylogenetic analysis demonstrates that 16S sequences of 29 OTUs and their close type strains share low identities (Table S6), which is below the threshold of 97% suggested for bacterial species delineation. It indicates that the strains represented by these OTUs might be potentially new species. Notably, OTUs 4, 6, 9, 13, 67 and 70 show low 16S sequence similarity (<91%) with *Eubostrichus topiarius* associated bacterium and uncultured Ectothiorhodospiraceae bacterium, and cluster into one clade within Gammaproteobacteria. Therefore, it is defined as Gammaproteobacteria un-group. Considering that the phylogenetic position of Gammaproteobacteria un-group (Fig. 5) is close to sulfur bacteria Ectothiorhodospiraceae and Thioglobaceae (Stackebrandt et al., 1984; Georgieva et al., 2020), Gammaproteobacteria un-group might be new bacteria group which can perform sulfur metabolism. However, in order to confirm novelity of the strains, whole genome sequences of these candidates and further experiments are required, such as by metagenome sequencing and fluorescence *in situ* hybridization.

### Habits influence on the bacterial communities

Although deep-sea hydrothermal vents and methane seeps are both chemosynthetic ecosystems, there are differences in the chemical compositions of the emitted fluid between the two environments (Scott & Fisher, 1995). In this study, it is revealed that ALMS has the highest overall microbe diversity among the three groups, while SLHV shows the lowest diversity. It may be due to the fact that seeps are milder and chemically more stable than hydrothermal vents acting as an essential driving force for more prosperous microbial communities in ALMS, while the transient nature of hydrothermal environments, temperature gradient, extremely high concentrations of sulfides, and heavy metals might be important contributors to the specificity of microbial composition in shrimp gill microbial communities, especially in SLHV close to the vent (Lietard & Pierre, 2009; Cruaud et al., 2017).

Determining the biogeochemical environment experienced by deep-sea organisms is not simple. By *in situ* detection, some environmental factors of the different sampling sites have been obtained (Fig. 1F, from Cao et al., 2021a and unpublished data by Cao et al.). In comparison, concentrations of H_2_S (2 mM) and CH_4_ (0.11 mM) and temperature (10.21 °C) are higher in the water column near the Iheya North hydrothermal vent where SLHV inhabits, while the content of dissolved oxygen (2.98 mg/L) and NO^3−^ (20.80–22.90 µM) in the water column of Formosa Ridge seep where ALMS lives is relatively high. In addition, the water of hydrothermal vent area with lower pH (5.20 and 5.50) are more acidic than those of the seep. In this study, it has been revealed that Campylobacteria and Gammaproteobacteria are the most dominant microbial community compositions of the alvinocaridid gills, consisting with other studies in crustaceans from vent and seep environments (Hügler et al., 2011; Sun et al., 2016; Zhang et al., 2018). Among them, Sulfurovaceae (all *Sulfurovum*) is ubiquitous in all samples, which is usually sulfur oxidizer and reducer and plays a vital role in the sulfur, nitrogen, hydrogen cycles, and even bio-fixation of CO_2_ in the deep sea (Mino et al., 2014; Kwon et al., 2015; Jeon et al., 2017). Despite these similarities, the relative abundances of main microbe compositions on gills of shrimps from different microevironments vary greatly, as suggested in studies for another alvinocaridid shrimp *R. exoculata* (Petersen et al., 2010; Durand et al., 2015).

Campylobacteria accounts for majority of the gill associated microbial community in SLHV close to the vent, including Sulfurovaceae (all *Sulfurovum*), and fewer Arcobacteraceae and Campylobacteraceae (*Campylobacter*) (Fig. 5; Table S4). It has been suggested that the deep-sea hydrothermal vent Campylobacteria are usually mesophilic to moderately thermophilic chemolitho-autotrophs capable of oxidizing hydrogen and sulfur compounds with nitrate, oxygen, and sulfur compounds as terminal electron acceptors (Campbell & Cary, 2001; Alain et al., 2002; Miroshnichenko et al., 2002; Inagaki et al., 2004; Nakagawa et al., 2005; Takai et al., 2005). The high proportions of S-trophic bacteria in SLHV are well in keeping with the extremely high concentration of H_2_S near the vent (2 mM) and relatively high temperature (10.21) (Fig. 1F). It also has been reported that Campylobacterota tends to occupy lower-oxygen habitats and use the reductive tricarboxylic acid (rTCA) cycle for carbon fixation (Meier et al., 2017). In hence, the extremely low oxygen (under the detection line) and reduced environment of hydrothermal vent should be also important factors in shaping the dominant position of Campylobacteria in gill associated bacterial community of shrimps near the vent.

Within a hydrothermal vent ecosystem, the chemical and thermal gradients generated by the mixing of hydrothermal fluid and sea water also provide a wide range of ecological niches for faunas and microbial communities living there (Takai & Nakamura , 2010). Within Iheya North hydrothermal vent area, ALHV living on the mussel beds away from the vent, occupies different ecological niches with SLHV. In the microbial community of ALHV, Sulfurovaceae (Campylobacteria) also takes the largest proportion, corresponding to the relatively higher concentration of reduced H_2_S (0.45 mM). However, more diverse sulfur oxidizer bacteria belonging to Gammaproteobacteria have been detected in ALHV, such as Gammaproteobacteria un-group and Thiotrichaceae (Goffredi, 2010; Lee et al., 2021). Rhodobacteraceae (Alphaproteobacteria), common marine heterotrophic bacteria with the ability to oxidize Fe (II), sulfur, ammonia and nitrite (Ehrenreich & Widdel, 1994; Straub, Rainey & Widdel, 1999; Miranda et al., 2021), are also identified in ALHV. It has been revealed that Gammaproteobacteria and Alphaproteobacteria, which typically dominate more oxic chemosynthetic habitats, commonly use the (Calvin-Benson-Bassham) CBB cycle for carbon fixation (Hügler & Sievert, 2011). Other microbes in ALHV, such as Rubritaleaceae (Verrucomicrobiae), are also obligatory aerobic chemoheterotrophic bacteria. Therefore, although no dissolved oxygen data is available, the result suggests that the concentration of dissolved oxygen in ALHV survival environment should be higher than that of the environment near the vent.

The shrimp *A. longirostris* inhabits both vent and seep environments. In ALMS group from Formosa Ridge methane seep in the South China Sea, Gammaproteobacteria is the dominant class in contrast with ALHV and SLHV from hydrothermal vent ecosystem. More diverse S-trophic Gammaproteobacteria are included, such as Gammaproteobacteria un-group, Thiotrichaceae, and Thioglobaceae. Moreover, *Methyloprofundus* and Marine Methylotrophic Group 2 (Methylomonadaceae) with significant capacity of anaerobic oxidation of methane (Martinez-Cruz et al., 2017) show higher relative abundance in comparison with those of SLHV and ALHV. Another methylotrophic bacteria, Marine Methylotrophic Group 3 (Methylophagaceae) are also detected in ALMS, some of which can utilize nitrate as denitrifiers (Villeneuve et al., 2013; Cao et al., 2021b). It has been reported that Methylomonadaceae usually co-occurs with denitrifiers and iron-cycling partners, which can effectively improve the ability of Methylomonadaceae to oxidize methane (Cabrol et al., 2020). The relatively higher abundance of Methylomonadaceae, Methylophagaceae and Rhodobacteraceae observed simultaneously in ALMS seems to support the view above. Reduced sulfur compounds and methane are the main energy sources to fuel the bacteria. It has been revealed that for host species in which methanotrophic and thiotrophic bacteria co-occur within the same individual, the relative abundance of these two bacteria is linked to the availability of sulfide and methane in their habitat (Colaço et al., 2011; Guezi et al., 2014). Therefore, although the concentration of methane in the water column where ALMS inhabit is not higher than those of SLHV environments, the relative lower concentration of H_2_S and higher concentration of nitrate and dissolved oxygen (Fig. 1F) in ALMS may ensure the flourish of methylotrophs and other diverse microbes.

### Hosts influence on the bacterial communities and core microbiome associated with alvinocaridid shrimps

Similar bacterial components have been found in the same alvinocaridid species *R. exoculata* that are geographically separated (Petersen et al., 2010), suggesting that alvinocaridid species may harbor certain species-specific microbiome regardless of their origins. In this study, despite striking environmental differences and distances (approximately 800 km) between the Iheya North hydrothermal vent and the Formosa Ridge seep in the South China Sea, analyses of PCoA and taxonomic classification show that ALMS and ALHV share more similar microbial compositions in comparison with that of SLHV. It indicates that there should be different selection patterns for epibiotic microbes in different species of shrimp hosts, and however, the inherent heterogeneity of bacteria in each environment cannot be ruled out. It suggests uptake of environmental bacteria might be a restricted process in *A. longirostris*. We may venture to assume that, if the *A. longirostris* individual from the Iheya North hydrothermal vent and the Formosa Ridge methane seep are swapped to each other’s habitats, the composition of the gill associated bacterial community of the “immigrants” will be updated to the same as that of the “natives” after some time under the premise of ensuring the survival of these individuals.

**Figure 6:**
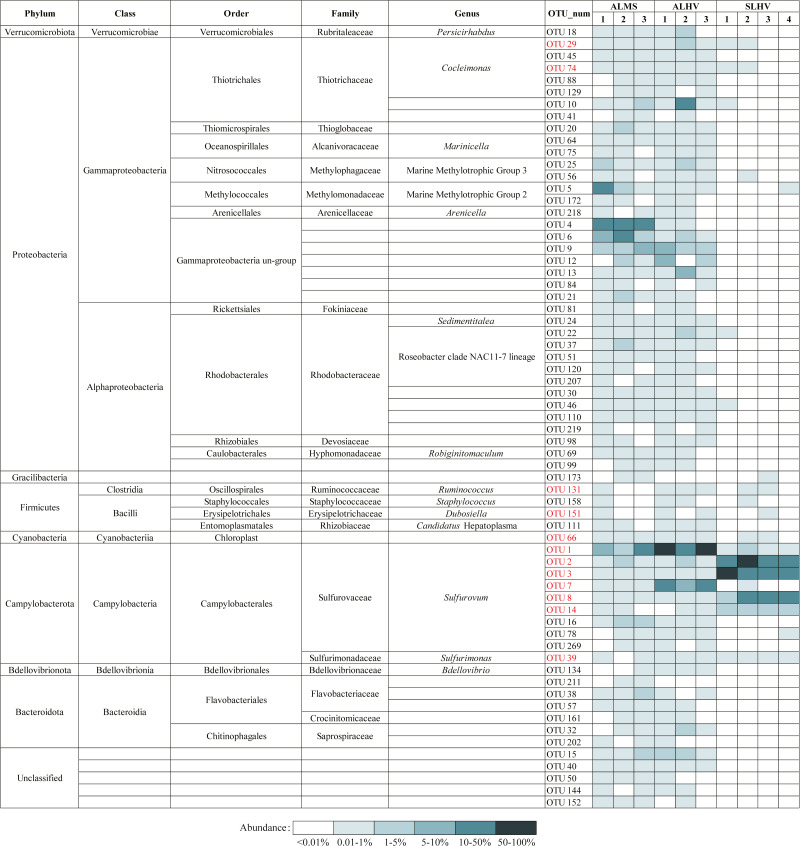
Bacteria present in *A. longirostris* core microbiome and their relative abundance in each sample. The OTUs in red represent bacteria in core microbiome of *A. longirostris* and *S. leurokolos*.

To further examine the conserved bacterial communities associated with *A. longirostris*, we have identified OTU represented bacterial species that are ubiquitously present in at least 50% of samples in ALMS and ALHV groups. A total of 65 species have been identified as candidates of the core microbiome in *A. longirostris*, with Gammaproteobacteria, Alphaproteobacteria and Campylobacteria constituting the greatest proportions of the putative core microbiome (33.85%, 21.54% and 15.38% respectively; Fig. 6). Bacteroidia, Bacilli and other minor groups are also included. In detail, Rhodobacteraceae (ten species), Sulfurovaceae (nine species), Thiotrichaceae (seven species), Gammaproteobacteria un-group (seven species), and Flavobacteriaceae (three species) families have the highest relative abundance present in the putative core microbiome (Fig. 6). We notice that most of the species identified in the *A. longirostris* core microbiome are present in low abundance. Only two species (OTU1: *Sulfurovum* and OTU9: Gammaproteobacteria un-group) have an average relative abundance higher than 2.90% within samples of each group, ALMS and ALHV. For example, Rhodobacteraceae (Alphaproteobacteria) shows lower relative abundance in ALMS and ALHV, but in the core microbiome harbored by *A. longirostris*, it takes the largest proportion. It indicates that bacteria that form stable and species-specific associations may be present at low relative abundance in the microbiome of alvinocaridid shrimps as revealed in the coral core bacterial communities (Pootakham et al., 2017).

Moreover, 11 species are conserved in ALMS, ALHV and SLHV, including seven species from Campylobacterota (six Sulfurovaceae, *Sulfurovum*, one Sulfurimonadaceae, *Sulfurimonas*), two Gammaproteobacteria (Thiotrichaceae, *Cocleimonas*), and two Firmicutes (one Ruminococcaceae, *Ruminococcus*, and one Erysipelotrichaceae, *Dubosiella*). A recent study has found that Gammaproteobacteria including Thiotrichaceae, can coevolve with their hosts, while Campylobacteria like Sulfurovaceae is globally distributed in marine sulfidic environments and has a weak host preference (Lee et al., 2021). This may partly explains why Sulfurovaceae accounts for a high proportion in the gill associated bacterial communities of the three groups of alvinocaridid shrimps, while Thiotrichaceae seem to mainly colonize only in *A. longirostris*. However, although 17 distinct bacterial 16S rRNA phylotypes of *Sulfurovum* are included in species constituting the dominant microbial community of the shrimps, OTUs of *Sulfurovum* branch two, such as OTU3 and OTU8, show high abundance in SLHV specifically (Fig. 5), which might be caused by the host selection or the result of co-evolution with the specific host or extreme vent ecological niches.

Therefore, variations in the community composition of gill associated bacteria in ALMS, ALHV, and SLHV are likely the result of a series of factors. The environmental physicochemical characteristics of the habitats and the host recognition have significant impact on the epibiotic community. Further studies concerning microbial diversity of liquid, rock, and sediment surface from the environments around these shrimps will benefit our understanding of whether the gill bacteria are an active embrace or not. Given that gill is one of the organs that directly exchange substances between shrimps and the external environment, we believe that the different bacterial communities found on shrimp gills in this study may play important roles in the adaptation to the extreme environments of the hosts. Further research based on metagenomics, metatranscriptomics and proteomics, as well as *in situ* experiments, are expected to provide deeper and novel insights into the microbe-host system in deep-sea chemosynthetic environments (Forget & Kim Juniper, 2013; Motoki et al., 2020).

## Conclusions

In the present work, full-length 16S rRNA sequences have been obtained to study the diversity and structure of the alvinocaridid shrimps’ gill associated bacterial communities in different deep-sea chemosynthetic environments. It has been found that the epibiotic bacteria community in each group closely corresponds to the physical and chemical factors of the shrimp’s habitat environment. Overall, Campylobacteria and Gammaproteobacteria are the dominant microbial community compositions of the alvinocaridid gills, involving sulfur and methane metabolism, denitrification and bio-fixation of CO_2_. In comparisopn, ALMS group has higher microbial diversity, and more Methylomonadaceae related to methane oxidation is detected in this group, while the bacterial community of SLHV is almost entirely composed of sulfur-oxidizing or reducing bacteria, Sulfurovaceae. More diverse sulfur metabolism-related bacteria species, such as Thiotrichaceae, Thioglobaceae and an untified Gammaproteobacteria, have been detected only in *A. longirostris* from MS and HV instead of *S. leurokolos*. All the results indicate that the epibiotic bacteria community of alvinocaridid shrimps might be co-determined by the living environments and the specific host selection. However, further genomics and experiments are required to investigate the interaction between the epitotic communities and the shrimp hosts.

##  Supplemental Information

10.7717/peerj.13758/supp-1 Supplemental Information 1 Sequence alignments of nine OTUs belonging to *Sulfurovum* The initiation of primer sequences usually used for amplifying different variation regions of 16S is marked with squares in different colors.

10.7717/peerj.13758/supp-2 Supplemental Information 2 Wilcoxon rank-sum test statistics of alpha-diversity between three groups *P* < 0.05 represents significant difference.* P* < 0.01 represents extremely significant difference.

10.7717/peerj.13758/supp-3 Supplemental Information 3 Permutational multivariate analysis of variance among different sample groups

10.7717/peerj.13758/supp-4 Supplemental Information 4 Number of species at different taxonomic levels in each sample

10.7717/peerj.13758/supp-5 Supplemental Information 5 Bacteria community composition and relative abundance at phylum and class levels

10.7717/peerj.13758/supp-6 Supplemental Information 6 Bacteria community composition and relative abundance at family and genus levels

10.7717/peerj.13758/supp-7 Supplemental Information 7 Summary of relative abundance of top 95% OTUs in ALMS, ALHV and SLHV, and the OTU sequence similarities with their relative close phylogenetic species

## Additional Information and Declarations

### Field Study Permissions

The following information was supplied relating to field study approvals (i.e., approving body and any reference numbers): Field experiments were approved by the Research Council of the Institute of Oceanology, Chinese Academy of Sciences (project number: QYZDB-SSW-DQC036).

### Data Availability

The following information was supplied regarding data availability: The raw data is available at NCBI SRA: PRJNA791844, and figshare: Wang, Aiyang (2021): gill symbiont of alvinocaridid shrimps16S rRNA gene. figshare. Dataset. https://doi.org/10.6084/m9.figshare.17696150.v1.
