# Supplementary figures and images for: Full-length 16S rRNA amplicon sequencing reveals the variation of epibiotic microbiota associated with two shrimp species of Alvinocarididae: possibly co-determined by environmental heterogeneity and specific recognition of hosts
Source: PeerJ. 2022 Aug 8;10:e13758. doi: 10.7717/peerj.13758 (PMC9368993; doi:10.7717/peerj.13758)

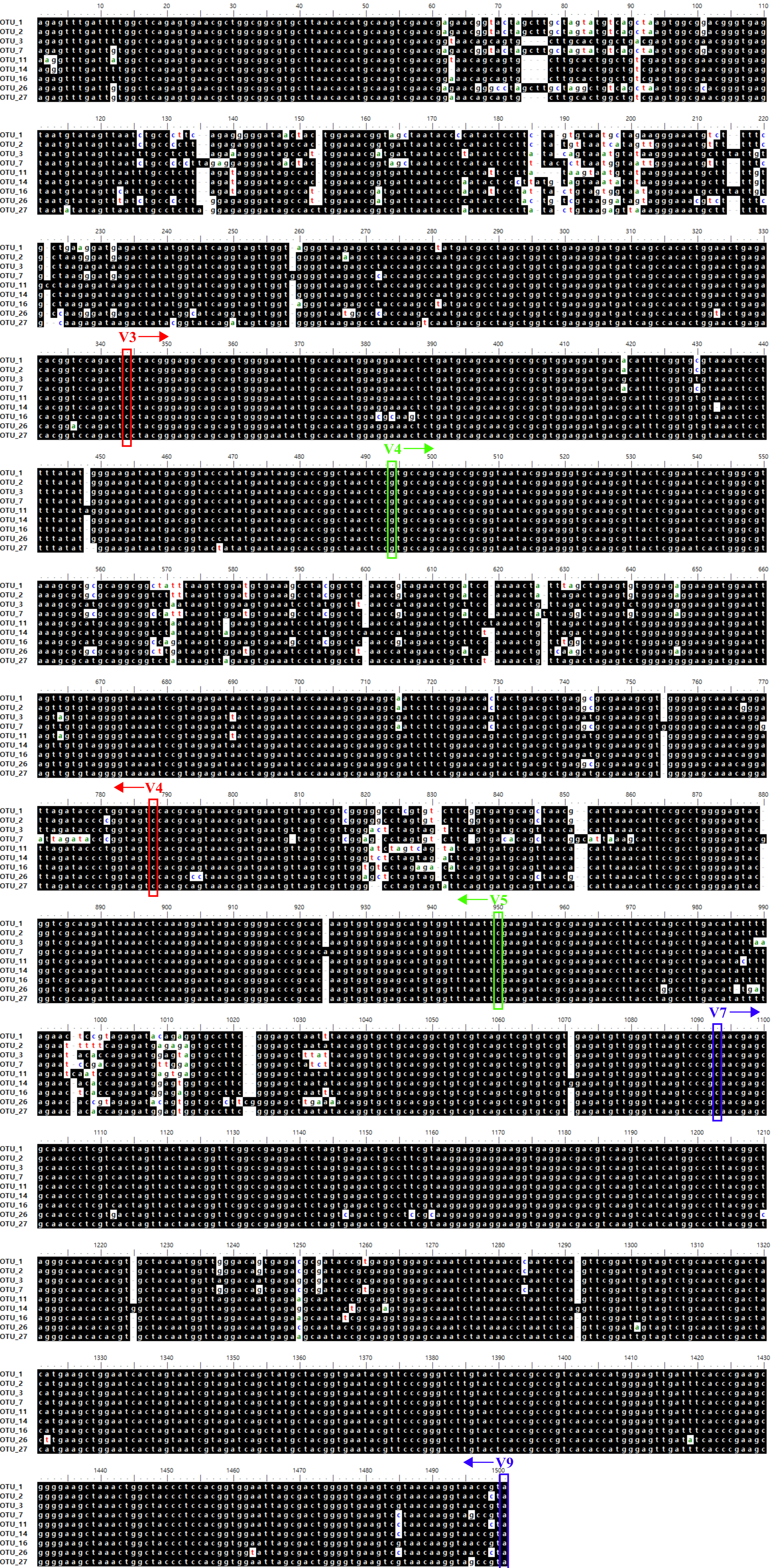

Supplement: Supplemental Information 1 — The initiation of primer sequences usually used for amplifying different variation regions of 16S is marked with squares in different colors. [file peerj-10-13758-s001.pdf]
