# Supplementary material for: Full-length 16S rRNA amplicon sequencing reveals the variation of epibiotic microbiota associated with two shrimp species of Alvinocarididae: possibly co-determined by environmental heterogeneity and specific recognition of hosts
Source: PeerJ. 2022 Aug 8;10:e13758. doi: 10.7717/peerj.13758 (PMC9368993; doi:10.7717/peerj.13758)
Supplement: Supplemental Information 2 — P < 0.05 represents significant difference. P < 0.01 represents extremely significant difference. [file peerj-10-13758-s002.docx]

**Table S1.**

**Wilcoxon rank-sum test statistics of alpha-diversity between three groups.** *P* < 0.05 represents significant difference. *P* < 0.01 represents extremely significant difference.

| Index type | ALHV vs ALMS  *P*-value | ALHV vs SLHV  *P*-value | ALMS vs SLHV  *P*-value |
| --- | --- | --- | --- |
| Chao1 | 0.2476 | 0.0119 | 0.0022 |
| Shannon | 0.1278 | 0.7470 | 0.0655 |
