# Supplementary material for: Full-length 16S rRNA amplicon sequencing reveals the variation of epibiotic microbiota associated with two shrimp species of Alvinocarididae: possibly co-determined by environmental heterogeneity and specific recognition of hosts
Source: PeerJ. 2022 Aug 8;10:e13758. doi: 10.7717/peerj.13758 (PMC9368993; doi:10.7717/peerj.13758)
Supplement: Supplemental Information 3 [file peerj-10-13758-s003.docx]

**Table S2.**

**Permutational multivariate analysis of variance among different sample groups.**

| Group | *R²*-value | *P*-value |
| --- | --- | --- |
| ALHV-ALMS | 0.53736 | 0.100 |
| ALHV-SLHV | 0.86160 | 0.037 |
| ALMS-SLHV | 0.73108 | 0.001 |
