# Supplementary material for: Full-length 16S rRNA amplicon sequencing reveals the variation of epibiotic microbiota associated with two shrimp species of Alvinocarididae: possibly co-determined by environmental heterogeneity and specific recognition of hosts
Source: PeerJ. 2022 Aug 8;10:e13758. doi: 10.7717/peerj.13758 (PMC9368993; doi:10.7717/peerj.13758)
Supplement: Supplemental Information 4 [file peerj-10-13758-s004.docx]

**Table S3.**

**Number of taxonomic units at different levels in each sample.**

| Sample | Phylum | Class | Order | Family | Genus |
| --- | --- | --- | --- | --- | --- |
| ALMS1 | 16 | 28 | 63 | 87 | 78 |
| ALMS2 | 14 | 18 | 38 | 45 | 32 |
| ALMS3 | 10 | 12 | 25 | 36 | 32 |
| ALHV1 | 12 | 17 | 36 | 36 | 30 |
| ALHV2 | 11 | 16 | 45 | 50 | 45 |
| ALHV3 | 13 | 16 | 32 | 37 | 34 |
| SLHV1 | 8 | 10 | 18 | 23 | 19 |
| SLHV2 | 11 | 15 | 31 | 38 | 36 |
| SLHV3 | 11 | 14 | 25 | 32 | 30 |
| SLHV4 | 7 | 8 | 16 | 20 | 18 |
| Total | 21 | 40 | 89 | 124 | 135 |
