# Supplementary material for: Full-length 16S rRNA amplicon sequencing reveals the variation of epibiotic microbiota associated with two shrimp species of Alvinocarididae: possibly co-determined by environmental heterogeneity and specific recognition of hosts
Source: PeerJ. 2022 Aug 8;10:e13758. doi: 10.7717/peerj.13758 (PMC9368993; doi:10.7717/peerj.13758)
Supplement: Supplemental Information 5 [file peerj-10-13758-s005.docx]

**Table S4.**

**Bacteria community composition and relative abundance at phylum and class levels.**

| Phylum | Abundance % | | | Class | Abundance % | | |
| --- | --- | --- | --- | --- | --- | --- | --- |
|  | ALMS | ALHV | SLHV |  | ALMS | ALHV | SLHV |
| Campylobacterota | 19.45 | 69.29 | 98.57 | Campylobacteria | 19.45 | 69.26 | 98.56 |
| Proteobacteria | 74.77 | 25.38 | 0.39 | Gammaproteobacteria | 70.58 | 23.34 | 0.30 |
| Verrucomicrobiota | 0.48 | 1.59 | 0.01 | Alphaproteobacteria | 4.14 | 2.05 | 0.08 |
| Bacteroidota | 1.80 | 1.10 | 0.45 | Verrucomicrobiae | 0.47 | 1.58 | 0.00 |
| Firmicutes | 0.25 | 0.43 | 0.22 | Bacteroidia | 1.78 | 1.11 | 0.46 |
| Bdellovibrionota | 0.23 | 0.03 | 0.00 | Bdellovibrionia | 0.19 | 0.03 | 0.00 |
| Cyanobacteria | 0.07 | 0.09 | 0.15 | Bacilli | 0.16 | 0.30 | 0.15 |
| Actinobacteriota | 0.16 | 0.04 | 0.04 | Cyanobacteriia | 0.07 | 0.09 | 0.15 |
| Gracilibacteria | 0.11 | 0.12 | 0.07 | Actinomycetia | 0.10 | 0.01 | 0.02 |
| Desulfobacterota | 0.19 | 0.00 | 0.01 | Desulfuromonadia | 0.10 | 0.00 | 0.00 |
| Others | 2.49 | 1.93 | 0.09 | Others | 2.96 | 2.23 | 0.28 |
