# Supplementary material for: Full-length 16S rRNA amplicon sequencing reveals the variation of epibiotic microbiota associated with two shrimp species of Alvinocarididae: possibly co-determined by environmental heterogeneity and specific recognition of hosts
Source: PeerJ. 2022 Aug 8;10:e13758. doi: 10.7717/peerj.13758 (PMC9368993; doi:10.7717/peerj.13758)
Supplement: Supplemental Information 6 [file peerj-10-13758-s006.docx]

**Table S5.**

**Bacteria community composition and relative abundance at family and genus level.**

| Family | Abundance (%) | | | Genus | Abundance (%) | | |
| --- | --- | --- | --- | --- | --- | --- | --- |
|  | ALMS | ALHV | SLHV |  | ALMS | ALHV | SLHV |
| Sulfurovaceae | 18.66 | 69.21 | 94.06 | Sulfurovum | 18.67 | 69.19 | 93.91 |
| Gammaproteobacteria un-group | 44.01 | 14.37 | 0.00 | Marine_Methylotrophic_Group_2 | 17.49 | 0.29 | 0.01 |
| Methylomonadaceae | 19.38 | 0.32 | 0.01 | Persicirhabdus | 0.40 | 1.53 | 0.00 |
| Thiotrichaceae | 1.77 | 6.77 | 0.16 | Marine_Methylotrophic_Group_3 | 1.11 | 0.60 | 0.00 |
| Rubritaleaceae | 0.40 | 1.57 | 0.00 | Sulfurimonas | 0.24 | 0.07 | 0.74 |
| Thioglobaceae | 1.69 | 0.27 | 0.00 | Campylobacter | 0.10 | 0.00 | 1.64 |
| Rhodobacteraceae | 3.27 | 1.70 | 0.04 | Roseobacter_clade_NAC11-7_lineage | 1.18 | 1.15 | 0.02 |
| Arcobacteraceae | 0.17 | 0.00 | 2.11 | Cocleimonas | 0.39 | 1.04 | 0.06 |
| Methylophagaceae | 1.10 | 0.60 | 0.00 | Methyloprofundus | 0.43 | 0.00 | 0.00 |
| Saprospiraceae | 0.64 | 0.78 | 0.01 | Sedimentitalea | 0.96 | 0.36 | 0.00 |
| Others | 8.91 | 4.41 | 3.61 | Others | 59.03 | 25.77 | 3.62 |
