# Supplementary material for: Full-length 16S rRNA amplicon sequencing reveals the variation of epibiotic microbiota associated with two shrimp species of Alvinocarididae: possibly co-determined by environmental heterogeneity and specific recognition of hosts
Source: PeerJ. 2022 Aug 8;10:e13758. doi: 10.7717/peerj.13758 (PMC9368993; doi:10.7717/peerj.13758)
Supplement: Supplemental Information 7 [file peerj-10-13758-s007.docx]

**Table S6.**

**Summary of** **relative abundance of top 95% OTUs in ALMS, ALHV and SLHV, and the OTU sequence similarities with their relative close phylogenetic species.**

| OTU | Subject ID | Identity (%) | Abundance % | | |
| --- | --- | --- | --- | --- | --- |
|  |  |  | ALMS | ALHV | SLHV |
| OTU_1 | KX097420.Unc05ubi | 99.02 | 14.62 | 54.68 | 0.65 |
| OTU_2 | KU942570.Unc2qsdx | 97.91 | 0.97 | 0.83 | 36.73 |
| OTU_3 | FJ497307.Unc005an | 98.04 | 0.33 | 0.13 | 35.44 |
| OTU_4 | GU145546.Unc15582 | 89.46 | 25.16 | 0.27 | 0.00 |
| OTU_5 | EU107483.UncMeth4 | 99.04 | 17.31 | 0.26 | 0.01 |
| OTU_6 | GU145546.Unc15582 | 89.13 | 14.92 | 0.84 | 0.00 |
| OTU_7 | AY197375.UncPr574 | 97.37 | 0.51 | 12.40 | 0.01 |
| OTU_8 | EU107475.UncSul26 | 98.33 | 0.10 | 0.09 | 9.52 |
| OTU_9 | HQ191085.UncSed93 | 90.17 | 2.93 | 4.99 | 0.00 |
| OTU_10 | EU107484.UncG1560 | 98.17 | 0.98 | 4.69 | 0.06 |
| OTU_11 | EU107475.UncSul26 | 97.63 | 0.05 | 0.00 | 4.29 |
| OTU_12 | AJ319041.EboTopi3 | 100.00 | 0.52 | 4.44 | 0.00 |
| OTU_13 | FN553591.UncSe104 | 88.92 | 0.14 | 3.71 | 0.00 |
| OTU_14 | EU107475.UncSul26 | 98.05 | 0.01 | 0.01 | 2.39 |
| OTU_15 | JQ287046.Unc20250 | 98.36 | 0.74 | 1.41 | 0.00 |
| OTU_16 | JQ287048.Unc66034 | 98.74 | 1.55 | 0.61 | 0.00 |
| OTU_17 | JQ287069.Unc59213 | 97.78 | 0.00 | 0.00 | 1.62 |
| OTU_18 | KT257814.Unc0541s | 97.00 | 0.39 | 1.53 | 0.00 |
| OTU_19 | KT257859.Unc0542n | 94.90 | 0.00 | 0.00 | 1.30 |
| OTU_20 | AB479078.B80Spec2 | 97.75 | 1.61 | 0.27 | 0.00 |
| OTU_21 | KR857623.Unc2ld9t | 89.57 | 1.66 | 0.09 | 0.00 |
| OTU_22 | JN874320.Unc007l9 | 97.00 | 0.50 | 0.85 | 0.02 |
| OTU_23 | FJ497323.Unc71625 | 97.61 | 1.43 | 0.00 | 0.00 |
| OTU_24 | JN873985.Unc00887 | 98.43 | 0.90 | 0.35 | 0.00 |
| OTU_25 | JF268399.Unc29844 | 98.56 | 0.81 | 0.42 | 0.00 |
| OTU_26 | FN658694.IBNProt2 | 98.05 | 0.00 | 0.00 | 0.92 |
| OTU_27 | EU107475.UncSul26 | 97.84 | 0.01 | 0.02 | 0.74 |
| OTU_28 | FN658699.GX3Pro47 | 92.29 | 0.04 | 0.70 | 0.00 |
| OTU_29 | KX581180.Unc2lhq3 | 99.45 | 0.14 | 0.57 | 0.01 |
| OTU_30 | JQ287083.Unc00c3y | 99.35 | 0.64 | 0.13 | 0.00 |
| OTU_31 | JQ287116.Unc08k8w | 98.12 | 0.01 | 0.00 | 0.60 |
| OTU_32 | JX017055.Unc47768 | 88.70 | 0.10 | 0.53 | 0.00 |
| OTU_33 | KJ603242.IBNProt3 | 97.00 | 0.00 | 0.00 | 0.53 |
| OTU_34 | FJ497307.Unc005an | 100.00 | 0.00 | 0.00 | 0.49 |
| OTU_35 | AM268719.UncPr563 | 97.00 | 0.00 | 0.00 | 0.43 |
| OTU_36 | EU107476.UncSul23 | 97.84 | 0.00 | 0.01 | 0.44 |
| OTU_37 | CP032125.Rc7Ba652 | 97.00 | 0.42 | 0.16 | 0.00 |
| OTU_38 | KT257761.Unc05441 | 97.79 | 0.46 | 0.04 | 0.00 |
| OTU_39 | EU265985.Unc60905 | 98.54 | 0.21 | 0.07 | 0.14 |
| OTU_40 | KT257854.Unc05417 | 97.00 | 0.13 | 0.36 | 0.00 |
| OTU_41 | EU107484.UncG1560 | 97.68 | 0.32 | 0.08 | 0.00 |
| OTU_42 | JQ287069.Unc59213 | 97.00 | 0.00 | 0.00 | 0.35 |
| OTU_43 | FJ497307.Unc005an | 97.00 | 0.00 | 0.00 | 0.31 |
| OTU_45 | KT257804.Unc053zs | 97.20 | 0.06 | 0.33 | 0.00 |
| OTU_46 | KC631560.Unc84122 | 97.00 | 0.36 | 0.02 | 0.01 |
| OTU_47 | GQ357038.Unc22295 | 91.05 | 0.00 | 0.34 | 0.00 |
| OTU_48 | FNVW02001120.HJ0Bac13 | 98.09 | 0.35 | 0.00 | 0.00 |
| OTU_49 | JN256000.UncEp113 | 98.11 | 0.27 | 0.00 | 0.00 |
| OTU_50 | EU265790.UncGa344 | 97.81 | 0.28 | 0.01 | 0.00 |
| OTU_51 | KM873092.UncPel35 | 97.00 | 0.19 | 0.09 | 0.00 |
| OTU_52 | JF344176.Unc05pbj | 92.79 | 0.26 | 0.00 | 0.00 |
| OTU_54 | LBBO01000216.GUQHVe17 | 92.43 | 0.23 | 0.00 | 0.00 |
| OTU_55 | JX441483.Unc05e31 | 94.49 | 0.24 | 0.00 | 0.00 |
| OTU_56 | DQ270606.Unc14127 | 97.13 | 0.05 | 0.18 | 0.00 |
| OTU_57 | KT257761.Unc05441 | 96.00 | 0.13 | 0.09 | 0.00 |
| OTU_59 | FN773278.FNWEn307 | 98.67 | 0.25 | 0.00 | 0.00 |
| OTU_61 | JX017055.Unc47768 | 89.66 | 0.02 | 0.16 | 0.00 |
| OTU_62 | AB680371.LcbHomo4 | 99.73 | 0.04 | 0.09 | 0.06 |
| OTU_64 | DQ270618.Unc19709 | 98.77 | 0.06 | 0.12 | 0.00 |
| OTU_66 | DQ226511.Mr4Indic | 99.86 | 0.05 | 0.07 | 0.04 |
| OTU_67 | KC682692.Unc09rku | 87.60 | 0.00 | 0.17 | 0.00 |
| OTU_68 | FN600353.HZYSymb3 | 90.95 | 0.17 | 0.00 | 0.00 |
| OTU_69 | KT257740.Unc05438 | 98.36 | 0.15 | 0.02 | 0.00 |
| OTU_70 | GU145546.Unc15582 | 88.90 | 0.15 | 0.00 | 0.00 |
| OTU_72 | KU942528.Unc2qscj | 97.91 | 0.16 | 0.00 | 0.00 |
| OTU_73 | JQED01000019.GDJPsyc2 | 99.59 | 0.15 | 0.00 | 0.00 |
| OTU_76 | JQ287046.Unc20250 | 97.00 | 0.15 | 0.00 | 0.00 |
| OTU_77 | AJ704666.UncG1005 | 95.39 | 0.13 | 0.00 | 0.00 |
| OTU_81 | JQ337869.Unc71644 | 99.65 | 0.12 | 0.01 | 0.00 |
| OTU_82 | KX177000.Unc2lfkw | 93.00 | 0.13 | 0.00 | 0.00 |
| OTU_83 | KC682522.Unc71699 | 98.53 | 0.00 | 0.11 | 0.00 |
| OTU_85 | JQ287087.Unc48751 | 97.86 | 0.12 | 0.00 | 0.00 |
| OTU_86 | JQ287093.Unc20252 | 97.00 | 0.00 | 0.10 | 0.00 |
| OTU_95 | CP012351.H1JAc402 | 99.86 | 0.08 | 0.00 | 0.00 |
| OTU_100 | JN662235.UncGa919 | 94.19 | 0.09 | 0.00 | 0.00 |
| OTU_102 | FJ717235.Unc10633 | 90.06 | 0.08 | 0.00 | 0.00 |
| OTU_103 | AJ704666.UncG1005 | 97.00 | 0.08 | 0.00 | 0.00 |
| OTU_110 | GQ903363.Unc00a8q | 97.11 | 0.06 | 0.02 | 0.00 |
| OTU_113 | KX097435.Unc05uer | 97.69 | 0.06 | 0.00 | 0.00 |
| OTU_116 | JQ515607.UncSoran | 95.07 | 0.07 | 0.00 | 0.00 |
| OTU_118 | CP023656.G8QHyd40 | 99.66 | 0.06 | 0.00 | 0.00 |
| OTU_121 | GU197434.UncGa770 | 95.49 | 0.07 | 0.00 | 0.00 |
| OTU_122 | EU287317.Unc19957 | 97.00 | 0.06 | 0.00 | 0.00 |
| OTU_123 | JQ287441.Unc52848 | 97.16 | 0.04 | 0.02 | 0.00 |
| OTU_125 | AY028220.UncVe199 | 97.81 | 0.07 | 0.00 | 0.00 |
| OTU_127 | FJ497601.Unc00b5h | 84.73 | 0.05 | 0.01 | 0.00 |
| OTU_130 | JF344176.Unc05pbj | 90.76 | 0.05 | 0.00 | 0.00 |
| OTU_136 | CP006992.HXVSp702 | 99.79 | 0.04 | 0.00 | 0.00 |
| OTU_139 | DQ055844.GDJAestu | 97.75 | 0.04 | 0.00 | 0.00 |
| OTU_141 | AM402955.HJ0Endos | 97.55 | 0.04 | 0.00 | 0.00 |
| OTU_150 | KX097714.Unc05ugy | 99.16 | 0.03 | 0.00 | 0.00 |
| OTU_156 | JX458402.HJLSpeci | 98.14 | 0.03 | 0.00 | 0.00 |
| OTU_159 | KX581191.Unc2lesh | 97.68 | 0.03 | 0.00 | 0.00 |
| OTU_165 | FNVW02001120.HJ0Bac13 | 98.35 | 0.03 | 0.00 | 0.00 |
| OTU_171 | CP019162.G1XS4323 | 99.93 | 0.03 | 0.00 | 0.00 |
| OTU_175 | FUWD013298441.GJ6ZZ576 | 95.69 | 0.03 | 0.00 | 0.00 |
| OTU_177 | CP017414.IIFMari6 | 93.18 | 0.03 | 0.00 | 0.00 |
| OTU_179 | LC068955.H0XBact5 | 90.78 | 0.03 | 0.00 | 0.00 |
| OTU_192 | CP012202.GX5Mac89 | 100.00 | 0.02 | 0.00 | 0.00 |
| OTU_200 | LT996885.GGKSpe90 | 99.80 | 0.03 | 0.00 | 0.00 |
| OTU_220 | KJ569656.Unc29097 | 90.00 | 0.02 | 0.00 | 0.00 |
